# On Menstruation, &c.

**Published:** 1842-10

**Authors:** 


### Art. V.

1. *De la Menstruation; faire connaitre l'influence que cette fonction exerce sur les Maladies, et celle qu'elle en recoit.* Par A. Brierre de Boismont, d.m. *Mémoire couronné, par l'Académie Royale de Médecine dans la Seance des* 17 *Décembre*, 1840. (*Mémoires de l'Académie Royale de Médecine.* Tome IX. 1841.)

*On Menstruation, &c.* By A. Brierre de Boismont, m.d. 4to, pp. 130.

2. *De la Menstruation considérée dans ses rapports Physiologiques et Pathologiques.* Par A. Brierre de Boismont, m.d. &c.—*Paris*, 1842. 8vo, pp. 560.

*On Menstruation, &c.* By A. Brierre de Boismont.—*Paris*, 1842.

These two works may be said to be the same, and yet different. The one first on the list formed a portion of the volume for 1841 of the Memoirs of the French Academy of Medicine, and is, doubtless, the identical memoir for which the prize was awarded. The octavo volume is a separate publication appearing at a more recent date, and is an amplification of the quarto memoir. As accounting for placing both at the head of this article, it is necessary to state, that all the first part of the article—the statistical part—was drawn up before the second publication reached our hands. The second part of the article—the pathological part—is based on the octavo volume.

STATISTICS OF MENSTRUATION. The author informs us that he derives his data respecting menstruation from the examination of 1200 females, in the upper, middle, and lower classes of society; and that these observations extend over a period of ten years: 830 of these cases were investigated by himself. He commences by endeavouring to establish the mean age at which menstruation makes its first appearance in the country, in the towns, and in the capital of France. 276 women supply the data of investigation, as regards the country; 214 belonging to the northern provinces, 57 to the central, and only 6 to the southern provinces. In ascertaining the mean age at which these 276 individuals menstruated for the first time, the author shows by the following table that it is about the age of fourteen years and ten months:

*Country.*

| Age of its first appearance. | No. of cases. | Age of its first appearance. | No. of cases. | Age of its first appearance. | No. of cases. |
|---|---|---|---|---|---|
| 7½ | 1 | 13 | 30 | 19 | 9 |
| 8 | 0 | 14 | 42 | 20 | 8 |
| 9 | 4 | 15 | 54 | 21 | 3 |
| 10 | 1 | 16 | 28 | 22 | 1 |
| 11 | 19 | 17 | 23 | | |
| 12 | 27 | 18 | 26 | | |
| | 52 | | 203 | | 21 = 276 |

*Towns.* His observations upon the first appearance of the catamenia among females residing in towns extend over 205 individuals; of whom 160 came from the north, 40 from the central portion, and 5 from the south of France. The mean age presents a slight difference to the previous scale. "This difference, although inconsiderable, ought to be noticed, as showing that the effects of the towns are beginning to manifest themselves." (p. 107.)

| Age of its first appearance. | No. of cases. | Age of its first appearance. | No. of cases. | Age of its first appearance. | No. of cases. |
|---|---|---|---|---|---|
| 9 | 1 | 14 | 33 | 19 | 7 |
| 10 | 6 | 15 | 29 | 20 | 11 |
| 11 | 21 | 16 | 20 | 21 | 1 |
| 12 | 21 | 17 | 24 | 22 | 1 |
| 13 | 18 | 18 | 11 | 23 | 1 |
| | 67 | | 117 | | 21 = 205 |

"Thus in the towns the mean age at which the catamenia appear is somewhat earlier than in the country. According to our observations, the extreme ages at which this important function shows itself are nine and twenty-three, embracing a period of fifteen years. But this extent of time, especially as regards the first number, must not be considered as the strict limits of the two extremes of this epoch, for it is by no means common to meet with young people who have menstruated at seven, eight, or even five years of age, of which we will give an instance."

The author has deduced his observations upon menstruation in the *capital* from 203 individuals; of whom four fifths were born in Paris; the others were natives of different parts of France, but had resided at least a year in Paris before the catamenia had appeared.

"Of these 203 females, 171 belonged to that class of people in narrow circumstances who had been compelled to seek assistance from the hospitals. The mean age of these 171 is fourteen years and a little more than ten months, which agrees almost exactly with the numbers deduced by MM. Marc Despine, and Bouchacourt. We are struck by the height of this number; for it appears that menstruation here is more tardy than in towns of the second magnitude, and even in the country. But this result, so different to what might have been expected, is only so in appearance, everything resolves itself into order when we analyse the elements of which this number is composed. There exists in large capitals a class of females which, from their manners, habits, mode of life, and organization, are distinct from the population from which they have sprung, and resemble, to a certain extent, the richer classes, from whom also they differ in other respects. Females of this class (which we have called '*metis*,' of civilization,) menstruate sooner than the others.

Age of the appearance of the Catamenia among the 171 females of the above class.

| | | | | | |
|---|---|---|---|---|---|
| 9 | 2 | 14 | 24 | 19 | 4 |
| 10 | 5 | 15 | 27 | 20 | 3 |
| 11 | 18 | 16 | 22 | 21 | 1 |
| 12 | 9 | 17 | 19 | 22 | 4 |
| 13 | 20 | 18 | 12 | 23 | 1 |
| | 54 | | 104 | | 13 = 171 |

"Hence it appears that the highest number is fifteen years, which scarcely differs from the preceding, since we may consider that the common age is comprised between the ages of fourteen and fifteen years."

Among the class of females which the author has denominated "*metis*,"* the mean age at which menstruation appears is somewhat lower than in the preceding class; and his observations on those belonging to the nobility, mercantile, and burger classes, show that the catamenia make their first appearance still earlier here. These investigations lead us to the following interesting results:

The mean age at which the catamenia make their first appearance—

| | | |
|---|---|---|
| In the poorer classes is | 14·842 or 14 years and 10 months. |
| In the class called "*metis*" | 14·405 or 14 ,, 5 ,, |
| In the richer classes | 13·660 or 13 ,, 8 ,, |

From the whole of his observations, as to the first appearance of menstruation in the capital among women of the three above-mentioned classes (being a total of 359), it results that the mean age is fourteen years six months. The author then gives us his grand total of the 1200 individuals on which his entire data were founded.

Table of the first appearance of menstruation in 1200 females.

| | | | | | |
|---|---|---|---|---|---|
| 5 years | 1 | 12 years | 105 | 19 years | 35 |
| 6 ,, | 0 | 13 ,, | 132 | 20 ,, | 30 |
| $7\frac{1}{2}$ ,, | 1 | 14 ,, | 194 | 21 ,, | 8 |
| 8 ,, | 2 | 15 ,, | 190 | 22 ,, | 8 |
| 9 ,, | 10 | 16 ,, | 141 | 23 ,, | 4 |
| 10 ,, | 29 | 17 ,, | 127 | | |
| 11 ,, | 93 | 18 ,, | 90 | | |
| | 136 | | 979 | | 85 = 1200 |

* Mulatto, or half-caste, also mongrel, i. e. *intermediate.*

The mean age in this table is 14·842.

"It is lower than that of Manchester, which is 15·191; and higher than that of Marseilles and Toulon, which give together 14·015. So that we see in this case that latitude produces a very sensible effect, since the difference as to the epoch of menstruation at Paris and at the two southern towns, taken together, amounts to about one year."

In summing up the principal points of the first chapter, M. B. de Boismont comes to the conclusion already deduced from common observation—

"That menstruation generally appears later in the country; that it appears earlier in the towns, especially in the manufacturing ones; but that the maximum in this respect is in the capital. Although this becomes in effect a law, there are numerous exceptions to it. Thus the lower classes of the population, exposed to all the privations in the train of misery, contain a large number of young girls in whom the catamenia appears very late. On the contrary, those young females whose habits and mode of life bring them under the class which we have called the *metis* of civilization manifest this function at an earlier age. But it is particularly among the young ladies of the wealthy classes, of the nobility, &c. that the menses are found to appear soonest." (p. 114.)

He then investigates the effects which difference of *temperament* has on the period at which the catamenia first appear. From the examination of 477 females of the lower classes, he shows that menstruation appears earliest in those of the sanguine temperament, viz. at 14·578; and latest in those of the lymphatic temperament, viz. 15·381.

In order to determine the effects of *constitution* upon this function, he divides 746 females into four classes or grades, beginning with those of a robust constitution and terminating with those who are delicate: the results show a marked difference as to the age at which the menses appear:

| | | | |
|---|---|---|---|
| "In the first division the mean age was observed to be | | | 14·520 |
| In the second | ,, | ,, | 14·706 |
| In the third | ,, | ,, | 14·807 |
| In the fourth | ,, | ,, | 15·470" (p. 116.) |

"The colour of the hair has also its influence, as well as the form of the body. Thus light hair and hair of a dark chesnut are found among women who menstruate late, whereas the brunettes are those in whom the menses appear earliest. Menstruation commences sooner in small women than in those who are tall." (p. 118.)

According to M. de Boismont's observations, the number of females in whom menstruation is accompanied or preceded by general or local symptoms is greater than of those in whom this is not the case, in the proportion of four and a fraction to one.

"The daytime seems to be the most favorable period for the appearance of the menses in females of a robust habit, or who take plenty of exercise; at least, it is during this period that we have observed menstruation appear most frequently in women of this class. This evidently results from walking, from their occupation, labour, exercise, and greater activity, which leads to the conclusion that well-regulated exercise must necessarily promote the coming on of the menses. In delicate women who use less exercise, and among those who menstruate easily, this function appears to occur more readily at night." (p.127.)

The author investigated the *duration of the menstrual period* in 562 females, with the following results:

| | | | |
|---|---|---|---|
| In 35 | cases the catamenia lasted | | 1 day. |
| 62 | ,, | ,, | 2 days. |
| 119 | ,, | ,, | 3 ,, |
| 78 | ,, | ,, | 4 ,, |
| 46 | ,, | ,, | 5 ,, |
| 21 | ,, | ,, | 6 ,, |
| 12 | ,, | ,, | 7 ,, |
| 172 | ,, | ,, | 8 ,, |
| 17 | ,, | ,, | 9, 10, and 15 days. |
| 562 | | | |

Hence it appears that the ordinary duration of the menses is comprised between one and eight days, and that the two periods during which the largest number of females menstruate are the eighth and the third days.

In examining the effects of marriage, pregnancy, parturition, and lactation upon the function of menstruation, we meet with a variety of results more or less interesting. Of 25 married women in whom menstruation had been irregular for some time, more or less, 14 became perfectly regular after marriage. The not uncommon appearance of the catamenia during pregnancy and lactation, so contrary to the old-established but erroneous law, has also been noticed by M. de Boismont:

"In 8 cases we have seen the menses appear during the two, three, and four first months after conception, in 3 instances they continued during the whole period of pregnancy. The same remarks are applicable to lactation; for if, in by far the majority of cases, the menses are completely suppressed, it is nevertheless true that they do appear under certain circumstances. Of 27 cases which presented anomalies of this nature, we noticed that they reappeared in 2 cases after six weeks of nursing, 4 times after four months, once after five, 3 times after nine, and once after eight months. In 12 cases they continued during the whole period of suckling. In the majority of these observations the health of the infant was not affected. In one instance, where the catamenia appeared regularly every three weeks during a nursing of twenty months' duration, the milk was like turbid water, and the weak, feeble child gradually sunk. Another female menstruated during eight pregnancies, but the milk was always serous and poor, and all her children died between the ages of four and five years." (p. 133.)

The effects of parturition upon catamenia are also well worthy of notice; and here again we have to thank the author for some highly valuable observations:

"Of 82 women who furnished us with distinct data as to the time at which the catamenia returned after labour, we may make the following classification:

| | | | |
|---|---|---|---|
| 1 | menstruated | almost immediately | after labour. |
| 1 | ,, | 8 days | ,, |
| 2 | ,, | 15 ,, | ,, |
| 4 | ,, | 3 weeks | ,, |
| 9 | ,, | 1 month | ,, |
| 38 | ,, | 6 weeks | ,, |
| 7 | ,, | 5 to 6 weeks | ,, |
| 7 | ,, | 2 months | ,, |
| 6 | ,, | 3 ,, | ,, |
| 2 | ,, | 4 ,, | ,, |
| 3 | ,, | 5 to 6 months | ,, |
| 2 | ,, | 7 to 8 ,, | ,, |
| 82 | | | |

"The period, therefore, from six weeks to two months, is that at which the catamenia most frequently reappear, but it ought not to excite surprise if they are suppressed for three or four months." (p. 134.)

We cannot agree with the author where he goes on to say that if the menses do not appear by this time, there is reason to fear some affection of the uterus and its appendages; because it is a well-known fact, at least in this country, that women not only frequently pass a much longer period without their reappearance, but even go from one pregnancy into another for several successive times, so that actually some *years* have intervened without any return of the catamenia, and yet without the least injurious effect on the woman's health.

A recent analysis of the menstrual fluid by M. Bouchardat has been quoted by the author. Great precaution seems to have been used in order to collect it pure. A speculum was closely fitted on to the cervix uteri, in order to prevent any admixture with the secretions of the vagina, and by keeping it applied for six hours, about an ounce of fluid was obtained. The results of M. Bouchardat's analysis are as follows:

| | |
|---|---|
| Water . . . | 90·08 |
| Fixed principles . | 6·92 |

The fixed principles consisted of—

| | |
|---|---|
| Fibrin, albumen, colouring matter | 75·27 |
| Extractive matter . . . | 0·42 |
| Fatty matter . . . . | 2·21 |
| Salts . . . . . . | 5·31 |
| Mucus . . . . . . | 16·79 |
| | 100·00 |

The elements of this analysis are identical with those of arterial blood. A portion of the same menstrual fluid was examined microscopically by M. Donné, who states that it consists—1st, of ordinary blood-globules, with their proper characters, in very considerable quantity; 2ndly, of mucus from the vagina, consisting of scales of epidermis furnished by the mucous membrane of the vagina; 3dly, of mucus-globules furnished by the cervix uteri.

The author confirms the observations of M. Nauche, viz. that the secretions of the vagina in a healthy person are uniformly acid. It is also acid in women after labour; it becomes alkaline when it is glairy, and is the product of inflammation; if the affection be confined to an isolated spot, the secretion from this spot is alkaline, while the other portions of the canal continue to secrete a matter essentially acid. From the consideration of the whole subject, M. Brierre de Boismont arrives at the following conclusions:

"That menstrual blood does not differ from arterial blood; that the varieties, as regards proportion, depend on circumstances connected with the individual; that the presence of blood-globules is incontestable; that the mucus which it has been supposed to contain, belongs to the uterus, its cervix, and to the vagina; that the acidity of the mucus accounts for the contradictory opinions of different authors." (p. 137.)

We confess ourselves disappointed to find that the *object* and *design* for which this function has been established in the human female have been so entirely lost sight of by the author. With such a mass of facts and observations bearing upon every feature of this function, we had hoped to have been favoured with many valuable and practical conclusions. He omits, or alludes very imperfectly to the fact, that diminution, irregularity or even suppression of the menses may occur without any disorder in the functions of the uterus, and may depend on a variety of causes, which act by lowering the tone and power of the system; in such cases the menses are, as it were, a safety-valve, and appear or not, according to the fulness or deficiency of its circulation. He does not seem aware that the catamenia constitute a discharge evidently designed to control the redundant supply of nutrition which is furnished during the generative life of the human female, when the powers of reproduction are at their highest degree of development. A mere accumulation of facts without a close and searching investigation of the circumstances under which they occurred, may be useful in a statistical point of view; but, practically speaking, they are of comparatively little value, because we are thus left in ignorance of the various causes which have determined the existence of these facts—the effects of them.

M. de Boismont has calculated the mean duration of the menstrual life of a woman from the results of 188 cases, and places it between twenty-eight and twenty-nine years, which does not differ materially from that which has been usually adopted, viz. thirty years.

The author's observations on leucorrhœa present nothing of particular interest: mere statistical details, without noting the state of the patients' health at the time are of little value, and lead to no practical results. From the reasons which we have already given, his summing up of the causes which influence the varieties of the catamenia, is extremely vague and unsatisfactory, and leads the reader to no clear or defined notions on the subject. Many facts of no peculiar novelty or rare occurrence are mentioned with an air of importance which would have led us to expect a few practical observations from the author in explanation, and which would doubtless have rendered their enumeration much more valuable. Thus he observes: "A great variety of circumstances may be observed in the same individual from the employment of medical treatment. We recollect the case of a lady in whom leeches were applied to the epigastrium, in consequence of an affection of that organ: the menses were immediately suppressed. The same person some years afterwards, being ill, had upwards of 160 leeches applied at different times, and, in spite of an enormous loss of blood, the menses at the next period showed no diminution of the ordinary quantity." (p. 156.) Without a single explanatory remark, such observations to a beginner are unintelligible; and to one already acquainted with the subject, they are both meager and valueless.

Some of the author's observations on dysmenorrhœa are sound and very deserving of notice:

"Dysmenorrhœa must ultimately induce that condition which will lay the foundation of uterine disease. We have seen where a profuse loss removed all the symptoms which it had occasioned. M. Lisfranc has well observed that the violent attacks of menstruation which constitute one form of dysmenorrhœa, after being continued for ten, fifteen, or twenty years, must necessarily produce a marked effect upon the uterus. He has convinced himself that painful menstruation is hereditary; and that if we ask patients suffering under this complaint, we shall find that other females of the same family have suffered in a similar way, and died of uterine disease." (p. 160.)

It is interesting to observe the effects of monastic life upon the menstruation, for it corresponds very closely with what we so frequently meet with in the boarding-schools of our own country. "It is seldom that we do not, after a few years, meet with a very considerable diminution in the quantity of catamenia," is the author's remark; we wish we could say that this was the only derangement which this function suffers by the worse than foolish system of education which is pursued in our fashionable institutions for female education. Few girls are many months at a boarding-school before the catamenia become sparing and irregular; with the due accompaniments of deranged digestion, unhealthy and constipated bowels, chilblains, and general loss of tone and vital energy, dysmenorrhœa, with all its terrible penalties, is but a too frequent result, and surely paves the way, if not to incurable barrenness, to frequent abortion, broken-down health, and ultimately organic disease. The general dyspepsy with more or less gastroenteric irritation and attendant leucorrhœa are so many effects of the same powerless and atonic state of the system, and have been duly noticed by the author. For the other effects of monastic life on the female he must speak himself:

"The critical age is seldom dangerous. Female recluses live a long time in a state of continued indisposition: a long life with habitual bad health is their lot. Cancer and also acute diseases are rare. Phthisis is very common. Death appears chiefly among patients of this class. The symptoms of phthisis may be carried to a great extent. Laennec has mentioned that in a convent where the discipline was very severe, the nuns and novices had the mind constantly directed to the punishments and retribution of another life; continued disappointments were instituted to curb the mind: suppression of the menses came on, and in two or three months afterwards symptoms of phthisis made their appearance. In a provincial town where we once resided a convent was established on a new system, the members of which, almost all of them young, distinguished themselves by their zeal and the rigid observation of their rules. In the course of one year ten of these young devotees had become victims to pulmonary consumption. Dysmenorrhœa, catamenial colics, hysteralgia of the same nature showed themselves during the early part of monastic seclusion. There were few of the recluses who had not some organic disease of the skin, alternating with lesions of the digestive functions and headach." (p. 164.)

The author enumerates several points of treatment, most of which we cannot but approve of, although we think he has omitted the most natural and important of all—fresh air and exercise.

"Rest, the horizontal posture, tranquillity of mind, and absence of every species of excitement are sufficient in a certain number of cases. If vascular congestion is evident, we must have recourse to abstraction of blood. When the nervous excitability is very distinct, the employment of antispasmodics and sedatives is indicated. Opium combined with diffusible stimuli has rendered great service in these cases. Laudanum in enemata has been very useful. We have seen in some nervous and lymphatic habits that hot wine with sugar has relieved these acute sufferings, as if by magic." (p. 165.)

A long and minute enumeration of the causes of chlorosis is of little use if not accompanied with sound and enlarged views of practical deductions based thereon. There can be no doubt but that chlorosis arises from general functional torpor and derangement, the result as well as the cause of feeble and irregular development. The powers of nutrition are nearly at a stand still, the blood is considerably altered in its qualities, and the whole system having sunk to the lowest stage of atony, makes but a fruitless struggle to maintain its various functions even in an imperfect state of action. As the author has referred to the Cyclopædia of Practical Medicine, we regret he has not benefited by the simple and highly practical articles on these subjects by Dr. Locock. We hardly agree with him when he states that dysmenorrhœa is less frequently met with in married women. In this country it is by no means unfrequently met with among married women, especially those of a gouty or rheumatic habit, where the uterine functions have been a good deal deranged by abortion.

In speaking of the influence which the appearance of the menses exerts upon existing diseases, the author observes: "Among the lesions which the catamenial period excites or tends to advance more rapidly, must be mentioned pulmonary consumption. We have observed it ten times, with a variety of symptoms. In every instance, with one exception, the disease was announced by hæmoptysis. . . . . . Many of these females informed us that they had constantly observed the attacks of hæmoptysis to be more constant and profuse at the menstrual periods, as well as before and after." (p. 189.)

M. de Boismont has proposed a question of deep interest, not merely in a statistical point of view, viz. What is the proportion of uterine affections among the deaths at the critical time of life? "We have heard," he says, "a celebrated professor state that he considered the number of females at Paris who were suffering under uterine diseases to be about 20,000. This estimate, not being founded on any precise statistical results, cannot be considered as very exact; but still it will not surprise the medical practitioner." (p. 192.)

How far this proportion is correct we have no means of proving: we can only state that the per centage of deaths among females in London from diseases of the generative organs is far beneath such an estimate. Thus, in the Third Annual Report of the Registrar-General for 1841, the mean annual mortality per cent. under the head of these diseases is only 0·46; and from a variety of circumstances, we have good reason to know that the proportion of uterine diseases in Paris far exceeds that in London. The following paragraph from the author not only in some measure explains this fact, but gives a lamentable view of the want of principle and morality of the French metropolis.

"Of 721 women who have formed the basis of these observations, 373 had had children, and 81 premature expulsion; so that, of the number of women delivered, a little more than a fourth part had not carried the produce of conception to the full time. Of these 81 cases, 25—nearly a third—might be considered as the results of abortion. This proportion is enormous; but yet it cannot excite surprise. From ignorance of moral principles, from want of good education, a number of young women suppose that they are committing no crime in destroying the existence of what they consider to be a shapeless and lifeless mass. Others under the influence of shame, prejudice, or naturally vicious, seek the assistance of that crowd of wretches who flourish in large towns. Misery, debauchery, certain positions in society also contribute to render the crime of infanticide more common." (p. 192.)

The influence of the "critical age" upon the life of females, is a question of much importance. If we were to pin our faith upon the popular and sometimes, too, professional belief, we should imagine it beset with dangers. But if we consult the best authorities, we find that which certainly agrees with our own observation, that the attendant hazards of this particular period are very much exaggerated. From the 43d degree of latitude to the 60th, says Benoiston de Chateâuneuf, that is to say, upon a line extending from Marseilles to St. Petersburg, passing by Vevai, Paris, Berlin, and Stockholm, we find, from the ages of thirty to seventy, no increase in the mortality of women but what naturally arises from the increase of years. The same inference follows from the experience of Muret de Vaud. Déparcieux,* too, comes to the same conclusion, and so does La Chaise.† It appears, indeed, from various statistical records, that the age of from forty to fifty is really more "critical" for men than women, whatever may be the kind of life the former may lead. "The question, then," says M. de Boismont, "appears to be definitively determined, that the 'critical' age does not increase the mortality of women." Still we may be allowed to add, it will always be "critical" to the ladies, in the most mortifying sense of the term; for though to them life may last, their beauty vanishes, or, at least, diminishes.

II. Pathology of Menstruation. Upon this part of the subject we shall refer to the octavo work, which enters rather more into details than the quarto memoir. And first, we must be allowed to touch briefly upon one or two points that are not adverted to by the author. We know no subject upon which medical practitioners are more closely pressed and questioned by females of all ranks, than upon this of menstruation; and none upon which young practitioners are more likely to betray their inexperience, especially to women of the upper classes. This may occur even in the mere mode in which enquiries are made by the practitioner respecting menstruation, or from his not comprehending the very equivocal hints which gentlewomen so frequently give as to the regular or irregular performance of the function. If, however, from want of professional association with this class of women, these hints are not at once understood; if the young practitioner, as we have often known to be the case, drives his patient to come directly to the point, which she would willingly avoid, the inference is immediately and not unjustly drawn, that he is a novice in the medical management of ladies of "gentle degree." For example: how often does a female, either from real or affected modesty, in answer to the very common question of, "Have you taken the medicine prescribed?" reply, "No; I did not think it would be right?" To the man of moderate experience this hint is sufficient. He knows at once that his patient is menstruating. But a tyro in such matters will not be at all instructed by this evasive answer, and will show by his manner that he knows not why his directions have not been obeyed. On the other hand, to females of the lower ranks, the medical enquirer will often find it necessary to put his questions in a very homely shape, or *they* will not understand *him*. Ask them "if they are *regular*," they answer, "Yes." "How often?" "Every morning," we have more than once known to be the reply, the question being supposed to refer to the state of the bowels. The young practitioner must also ever bear in mind that whatever may be the disease or ailment under which women are labouring, they are always strongly inclined to consider it as the effect of even the slightest irregularity of the menstrual

* Essai sur les Probabilités de la Vie humaine. † Topographie médicale de Paris.

function. And although such an opinion is, for the most part, founded on prejudice and imperfect observation, it is one not slightly to be set aside in practice.

Trite as may be the first remark the author makes upon *amenorrhœa*, it conveys an important truth that is too frequently forgotten. When a young female has arrived at the age of puberty, as a general rule, menstruation takes place. But this is not always the case. And if her health does not suffer, active medical interference is improper. But how often are mothers very anxious, and how often does the practitioner very improperly comply with their wishes, for the administration of some of the tribe of "forcing" remedies, when the girl, though a woman in age, is still a child in form and general development. Nothing can be more injudicious than this too common practical error.

The general description M. de Boismont gives of primary amenorrhœa claims no particular notice. We may observe that Morgagni mentions several cases of females who died without having ever menstruated, and in whom the uterus was found remarkably small and undeveloped. We have now before us a specimen of this kind. The uterus, taken from a young lady of sixteen years of age, is quite in a rudimentary state: not larger than that of a female of five years. She had not menstruated. How much mischief would be done in such cases by emmenagogue or stimulating remedies. We cannot, during life, detect this retarded development; but our practice would be guided by the childish or "backward" appearance of the patient, even though she had arrived at the age of puberty.

"Blueness of the skin," says the author, "was observed in two cases of suppression of the menses." Cases are related by Leutin, Trotter, and Marcet, in which a blue colour of the skin appeared suddenly, in a single day, after a sudden and complete suppression of the menses; the discoloration remained for six weeks, at the end of which time the patient died. A case of a similar nature was seen by Marc and others. In the first case we refer to there was no organic mischief of the heart or the respiratory apparatus. In neither of the cases was there any preternatural communication between the arteries and veins. The author suggests that this discoloration of the skin takes place in the same manner as in asphyxia, or after narcotic poisoning, as by opium. It must be confessed, we believe, that we cannot, as yet, explain what is the modification that the vital action undergoes in such instances. "Certain medicines sometimes cause suppression of the menses. We have several times known amenorrhœa to occur after the administration of copaiba and cubebs." (p. 306.) "It is often very difficult to ascertain the cause of suppression. In 30 cases we could not detect it." (p. 337.)

The account of *dysmenorrhœa* will be regarded as meager and unsatisfactory by any one acquainted with English writers on the same subject. We have upon a former occasion (Br. and For. Med. Rev., vol. II., p. 180,) offered a few remarks upon the treatment of this disease, which further experience has corroborated.

The description of *chlorosis* is respectable, and does not materially differ from that of Dr. Ashwell, (Br. and Fr. Med. Rev., vol. II., p. 175,) and other writers. We cannot say, however, that sufficient precision marks the directions as to the treatment to render them useful guides to those who require help from others. Several facts mentioned by the author confirm the pathological views of the nature of chlorosis, to which we have formerly adverted. (Br. and Fr. Med. Rev., vol. II., p. 176.) "The grounds for considering chlorosis a general affection, dependant upon a change in the quantity or qualities of the blood, are striking." In the ordinary state, the proportion of the serum to the coagulum of the blood is as 5 to 8; this proportion of these constituents varies, however, with many individual peculiarities. M. Jolly* has not seen a single case of chlorosis or anemia in which the proportion of serum did not exceed seven tenths of the whole mass of blood. In one instance it was nearly nine tenths. Still, however, M. de Boismont is inclined to the opinion that chlorosis is sometimes a local disease; that it reacts upon the general economy. He founds his belief upon the following facts: Practical experience affords us unequivocal evidence that in certain young females, menstruation is solicited in vain by tonics, steel medicines, or emmenagogues, and that it occurs after marriage or pregnancy. In his general remarks upon the influence of these occurrences in the physiological part of the work he has related some cases in point. Frank relates similar examples. The direct stimulation of the genital organs suffices to dispel the symptoms. Young females with all the signs of chlorosis are cured by sexual intercourse, without any medical agent. Chlorosis sometimes exists in women who are perfectly "regular." M. de Boismont gives examples of this rather unusual occurrence, for as a very general fact, it may be asserted, that if the chlorotic state be well marked, the menstrual function is either not performed at all, or at least very imperfectly. Difficulties, too, present themselves when we enquire into the nature of this disease. Is it always an asthenic disease? Ought it to be regarded as a variety of anemia? In the present state of our knowledge, the author is of opinion that we cannot assert that it is always asthenic. He has seen young women with all the symptoms of the disease, in whom the colour of the skin, the constitution, and temperament all indicated strength and power. Such a form of the malady has not escaped the attention of other physicians. Frank very confidently admits a species of sthenic chlorosis. Professor Wendt, of Breslau, has denominated this species by the title of "*chlorosis florida seu fortiorum.*"† Stoll, too, observed cases of chlorosis from plethora, which were very distinct from the ordinary species, and occurring in country girls accustomed to hard labour. Hoffman makes the same remark, and he occasionally had recourse to bleeding. Mason Good,‡ too, we may observe, divides chlorosis into two species, the atonic and entonic. Dr. Copland, we think, very correctly comments§ upon these attempts to establish this "sthenic or entonic" form of chlorosis in these terms: "This is an unnecessary refinement, no phenomena which warrant such a distinction presenting themselves in practice. Indeed, the entonic only consists of a state relatively of less deficiency of vital power than the atonic, and is in many cases merely the first stage of the disease; particularly when it occurs in tolerably strong females, and whilst the torpid function has not as yet extended much further than the sexual organs in which it originated, the

* Jolly. Du siege, de la nature et du traitement de la chlorose. Rev. Méd. Dec. 1839.
† Rust's Magazine, vol. xlv. ‡ Study of Medicine. 2d edit. vol. v. p. 110.
§ Dictionary of Practical Medicine. Part i. p. 315.

digestive, assimilating, and vascular organs not having sustained much disorder." Of the truth and practical importance of these remarks we have no doubt, and sure we are that if in this "entonic form" of the disease we come to the conclusion, that "copious and not unfrequently repeated venesections are necessary,"* we shall very quickly have cause to regret our departure from the more commonly received notions of the nature of the disease and the treatment it requires. In a word, we shall by this debilitating treatment hasten the disposition that exists in all such cases to the establishment of a state of complete atony, with cold and perhaps œdematous extremities, and a crowd of other evils, that every practitioner of moderate experience must know to be the usual concomitants of the chlorotic condition, when it has lasted long enough to exhaust the sum of vital power that may and nobody denies does exist at the very beginning of the disease. M. de Boismont is quite right in deprecating the belief that steel or any other remedy will cure chlorosis without pure air, wholesome food, tranquillity of mind, regular exercise, and other obvious and appropriate auxiliaries. We must not be too rigid in the dietetic management of chlorotic girls. Capriciousness of appetite is a marked feature of the disease. Very often it is difficult to induce them to eat at all, and they may be fairly and properly allowed to indulge their taste within reasonable limits, even if the articles of food they fancy should be opposed to our general principles.

The chapter "de la déviation des regles," *vicarious menstruation*, contains several remarkable cases. Examples of such deviations from the ordinary course of nature are far too common and well known to induce us to dwell long upon the subject. We are tempted, however, to give the following very striking case. We have before merely referred to it (vol. II. p. 180). Gardien† quotes the same case nearly word for word from M. Brulé. Our author gives it from the *Médecine Pratique* of Pinel:

"Miss A. had been subject to attacks of hysteria from the age of eleven, which were followed by vomiting of blood. She menstruated at fourteen; her health was re-established, and the catamenia continued to flow regularly for several months. A sudden fright suppressed the menses, and again hysteria came on. Vicarious menstruation now occurred. The legs swelled and were covered with vesicles, and during six months blood was regularly discharged from them. The left arm swelled and the legs recovered, and for a year there was a regular sanguineous discharge from the arm. A third deviation occurred from the left thumb which had been slightly wounded. The 'menses' flowed from this opening for six months. In the fourth year, two wounds were formed on the face, from an attack of erysipelas; one upon the side of the nose, the other on the upper eyelid. For two years the periodic discharge took place from these openings, and it no longer occurred from the thumb. The abdomen, in its turn, was attacked with erysipelas, and for five months regularly there was a discharge from the navel at each menstrual period. For four months the discharge proceeded from the inner ankle of the left foot; for two months from the left ear; for three from the left nipple. When the discharge did not flow from any one part, bleedings at the nose, and vomitings of blood, preceded by convulsions, pains in the head, and giddiness took place. After remaining some time at the Saltpêtrière, the health of this young female improved, and regular menstruation was established.—In another instance the young lady's

* Mason Good, loc. cit. p. 111.

† Traité complet d'Accouchemens, t. i. p. 238. 3me ed.

nose itched very much; it swelled and became red, and for several years blood was discharged from the nostrils regularly every month."

The circumstances of this case were accurately noted by Dr. Piedagnel. These examples are sufficient to instruct those who may not have seen instances of vicarious menstruation, of the curious deviations that occasionally occur. Spitting and vomiting of blood are more common, and once more, in reference to the latter kind of vicarious menstruation, we venture to quote from ourselves, to keep in mind the fact, that harmless as such cases in general are, they have been very unexpectedly fatal. (Br. and For. Med. Review, vol. II. p. 180.) We shall never forget the deep impression that the case here referred to made upon our minds, or the surprise of the learned physician who attended with us, when we informed him of the sudden and unlooked-for death of our patient. It is worthy of observation that sometimes vicarious menstruation occurs without any premonitory symptoms; sometimes, according to M. de Boismont, well-marked and very alarming symptoms precede it. Occasionally, too, a periodical diarrhœa has superseded the menstrual discharge during the whole life of the female.* We cannot quit this subject without one more remark. As a general rule we ought not to suppress, even if we can, the vicarious discharge, unless the natural flow of the catamenia is established. If we do arrest the unusual discharge from any other part, we are not certain the natural discharge may take place from the uterus, and thus we should expose the patient to the double danger of suppression of the former and possibly also of the latter. In all such cases we must act upon general principles, and endeavour, by the well known and general means, to establish the normal performance of the function of the uterus.

The subject of *menorrhagia* is dismissed too briefly by the author, considering the title of his work.

Under the head of *the influence of the first menstruation* upon disease, some rather interesting cases are mentioned. As for example, the cessation of violent and general chorea when the menses appeared.

The statistical tables, too, upon the frequency with which cancer of the uterus and polypi occur at the critical age of women are worth consulting. The chapter on the influence of menstruation on acute, chronic, and surgical diseases may be safely passed over. It deals in loose and unsatisfactory generalities.

M. de Boismont has collected with a good deal of care, all, and that does not amount to much, that is practically important respecting the influence of disease on menstruation. Louis, in his researches on Pulmonary Consumption, has noticed the influence of this disease on the menstrual discharge. Esquirol, too, has made some interesting remarks upon the same subject in reference to insanity. We have been recently informed by the physician of a large lunatic asylum that, as a general rule, menstruation is either suppressed or goes on very irregularly in insane females. Desormeaux touches upon the same subject. (Menstruation. Dict. de Med. 2me ed.) The Polish physicians state that during the existence of "Plica" the periodical discharge is frequently suspended, and that its return indicates the cure of the disease of the hair. Irritation of the nipple, from suction of the infant, causes in many women a

* Baudelocque. Art des Accouchemens, t. i. p. 185.

flow of the menses at irregular periods. "It may also cause a prolongation of the flow, in a word, true hemorrhage." (p. 469.) This statement puts us in mind of a remark made by Dr. Rigby, (System of Midwifery, p. 217,) when discussing the treatment of hemorrhage after labour. We would still continue the note of interrogation which we marked in the margin of the book at the time we read the following passage: "Where every means has failed to induce a sufficient or permanent degree of contraction, we believe that the only certain means which remains is putting the child to its mother's breast." This is strongly expressed.

After a return of the menses, any disease under which the patient may be labouring may still run a chronic and even incurable course.

The reputation that M. Brierre de Boismont enjoys as a very practised and practical writer cannot fail to be increased by the publication of this work. In a physiological point of view it is extremely interesting and instructive. By the assistance of his statistical tables he establishes many facts connected with the very important function of menstruation, in a much more striking and impressive manner than had previously been effected by preceding writers. The pathological part is decidedly inferior to the physiological. As a mere sketch it is unobjectionable; but it wants much to entitle it to rank as a complete or satisfactory treatise.

---

### Art. VI.

*Transactions of the Provincial Medical and Surgical Association.* Vol. X.—*London*, 1842. 8vo, pp. 354.

